# A clinical case of X-linked hypohidrotic ectodermal dysplasia caused by an Xq13.1 duplication affecting the EDA gene

**DOI:** 10.18699/vjgb-26-95

**Published:** 2026-10

**Authors:** V.I. Stepanenko, I.Zh. Zhalsanova, E.A. Fonova, A.S. Zuev, S.N. Gosudarkina, T.N. Kireeva, E.G. Ravzhaeva, A.A. Agafonova, G.N. Seitova, A.S. Monakhova, N.A Skryabin

**Affiliations:** Research Institute of Medical Genetics, Tomsk National Research Medical Center of the Russian Academy of Sciences, Tomsk, Russia; Research Institute of Medical Genetics, Tomsk National Research Medical Center of the Russian Academy of Sciences, Tomsk, Russia; Research Institute of Medical Genetics, Tomsk National Research Medical Center of the Russian Academy of Sciences, Tomsk, Russia; Research Institute of Medical Genetics, Tomsk National Research Medical Center of the Russian Academy of Sciences, Tomsk, Russia; Research Institute of Medical Genetics, Tomsk National Research Medical Center of the Russian Academy of Sciences, Tomsk, Russia; Research Institute of Medical Genetics, Tomsk National Research Medical Center of the Russian Academy of Sciences, Tomsk, Russia; Research Institute of Medical Genetics, Tomsk National Research Medical Center of the Russian Academy of Sciences, Tomsk, Russia; Research Institute of Medical Genetics, Tomsk National Research Medical Center of the Russian Academy of Sciences, Tomsk, Russia; Research Institute of Medical Genetics, Tomsk National Research Medical Center of the Russian Academy of Sciences, Tomsk, Russia; LLC “Biotech Campus”, Moscow, Russia; Research Institute of Medical Genetics, Tomsk National Research Medical Center of the Russian Academy of Sciences, Tomsk, Russia

**Keywords:** ectodermal dysplasia, hypohidrotic, Christ–Siemens–Touraine syndrome, EDA, copy number variations (CNV), next-generation sequencing (NGS), эктодермальная дисплазия, гипогидротическая дисплазия, синдром Криста–Сименса–Турена, EDA, вариации числа копий участков ДНК (CNV), массовое параллельное секвенирование (МПС)

## Abstract

X-linked hypohidrotic ectodermal dysplasia (XLHED; Christ–Siemens–Touraine syndrome, ICD-10: Q82.4) is the most common type of ectodermal dysplasia, caused by genetic disorders of ectodermal derivatives. The condition significantly impacts patients’ quality of life, presenting with a classic triad of hypohidrosis, hypotrichosis and hypodontia, which creates a risk of impaired thermoregulation and necessitates complex dental rehabilitation. The molecular basis of the disease lies in alterations of the EDA gene, which encodes ectodysplasin A. This protein belongs to the tumor necrosis factor (TNF) family and plays a critical signaling role in ectodermal-mesenchymal interactions during embryonic development. The present case report describes a boy with an XLHED phenotype caused by a previously unreported structural rearrangement. Whole-genome sequencing (WGS) identified a ~397.3 kb microduplication at the Xq13.1 locus directly involving the EDA gene. Comprehensive bioinformatic analysis ruled out additional pathogenic or likely pathogenic variants in other genes that could account for the patient’s phenotype. The Xq13.1 microduplication was confirmed in both the proband and his mother using quantitative real-time PCR (qPCR). Androgen receptor (AR) gene methylation analysis in the mother revealed a random X-chromosome inactivation (74/26 %), explaining her asymptomatic carrier status. The proband’s clinical manifestations were also compared to those of literature-reported patients with overlapping genomic rearrangements. Based on international and Russian guidelines for the interpretation of massively parallel sequencing data, the identified variant was classified as pathogenic. Identification of this structural rearrangement expands the known mutational spectrum of the EDA gene. Establishing a precise molecular genetic diagnosis in this family is critical not only for disease verification but also for optimizing patient management through a multidisciplinary approach. Furthermore, it enables comprehensive genetic counseling, identification of female carriers among relatives and the development of timely primary and secondary prevention measures for the disease in this family.

## Abstract (ru)

X-сцепленная гипогидротическая эктодермальная дисплазия (синдром Криста–Сименса–Турена, МКБ-10: Q82.4) представляет собой наиболее распространенную форму эктодермальной дисплазии, вызванной наследственными нарушениями развития производных эктодермы. Заболевание существенно влияет на качество жизни пациентов, проявляясь классической триадой симптомов: гипогидрозом, гипотрихозом и гиподонтией, что создает риски нарушения терморегуляции и требует сложной стоматологической реабилитации. Молекулярной основой патологии служат нарушения в гене EDA, ответственном за синтез эктодисплазина-А. Данный белок принадлежит к семейству фактора некроза опухоли (TNF) и играет критическую сигнальную роль в процессе эктодермально-мезенхимальных взаимодействий во время эмбрионального развития. В настоящей работе описан клинический случай мальчика с фенотипом X-сцепленной гипогидротической эктодермальной дисплазии, обусловленный ранее не описанной структурной перестройкой. Методом полногеномного секвенирования (WGS) была выявлена микродупликация локуса Xq13.1 размером приблизительно 397.3 Кб, непосредственно затрагивающая ген EDA. Комплексный биоинформатический анализ подтвердил отсутствие дополнительных патогенных или вероятно патогенных вариантов в других генах, которые могли обусловливать фенотип пациента. Микродупликация Xq13.1 подтверждена у пробанда и его матери методом количественной полимеразной цепной реакции в режиме реального времени. Анализ метилирования гена AR продемонстрировал у матери равновероятную инактивацию X-хромосомы (74/26 %), что объясняет ее бессимптомное носительство. Проведено сравнение клинических проявлений пробанда с описанными в литературе пациентами, имеющими близкие по координатам структурные перестройки. На основании международных и российских руководств по интерпретации данных массового параллельного секвенирования обнаруженный вариант классифицирован как патогенный. Идентификация этой структурной перестройки расширяет известный мутационный спектр гена EDA. Уточнение молекулярно-генетического диагноза в описанной семье имеет критическое значение не только для верификации нозологии, но и для коррекции тактики ведения пациента, включая междисциплинарный подход к лечению. Кроме того, это позволяет провести качественное медико-генетическое консультирование, выявить носительство у родственников женского пола и разработать эффективные меры первичной и вторичной профилактики заболевания в данной семье.

## Introduction

Ectodermal dysplasia is a genetically and clinically heterogeneous group of disorders characterized by defects in two or more ectodermal derivatives, including sweat glands, hair, and teeth (Visinoni et al., 2009). Hypohidrotic ectodermal dysplasia is a subgroup of ectodermal dysplasias, whose characteristic clinical feature is reduced sweat gland function, leading to impaired thermoregulation (Anbouba et al., 2020; Wohlfart et al., 2020). Pathogenic variants in four genes account for approximately 90 % of hypohidrotic ectodermal dysplasia cases: EDA (X-linked forms), EDAR and EDARADD (responsible for both autosomal dominant and autosomal recessive forms), and WNT10A (autosomal recessive forms) (Cluzeau et al., 2011).

X-linked hypohidrotic ectodermal dysplasia (Christ–Siemens– Touraine syndrome, XLHED) (OMIM: #305100) is a hereditary disorder affecting structures of ectodermal origin, such as hair, teeth, nails, and sweat glands. The disease is caused by pathogenic variants in the EDA gene, responsible for the synthesis of the protein ectodysplasin A. This protein belongs to the tumor necrosis factor (TNF) family of ligands and plays an important role in the formation of ectodermderived structures during embryogenesis (Kere et al., 1996; Mikkola, Thesleff, 2003). According to the literature, X-linked hypohidrotic ectodermal dysplasia is the most common form of hypohidrotic ectodermal dysplasia worldwide (Lamartine, 2003). The prevalence of this disease is estimated to range from 1 to 22 per 100,000 individuals (Cañueto et al., 2011; Nguyen-Nielsen et al., 2013). The clinical presentation of this disease involves various combinations of the following features: hypohidrosis, hypotrichosis, and hypodontia (Itin, Fistarol, 2004; Bagdey et al., 2022). This paper presents a clinical case involving an Xq13.1 duplication not described hitherto.

## Materials and methods

A family with a suspected case of ectodermal dysplasia in the son was enrolled in this study. The research was conducted in compliance with ethical standards as outlined in the World Medical Association’s Declaration of Helsinki. Written informed consent was obtained from all participating family members. The study protocol was approved by the Biomedical Ethics Committee of the Research Institute of Medical Genetics, Tomsk National Research Medical Center (Protocol No. 10, dated January 20, 2021).

Peripheral blood samples were collected from the patient and his parents into EDTA-containing tubes for molecular genetic analysis. Genomic DNA was extracted from whole blood using the standard phenol-chloroform method.

Whole-genome sequencing of the proband was performed within the collaborative research program with the National Genetic Initiative “100,000+Me” at Biotech Campus Company. Whole-genome sequencing was carried out on DNBSEQ-T7 platforms (MGI) using the MGIEasy FS PCR-Free Library Prep Set (MGI) for library preparation, producing 2 × 150 bp paired-end reads. The bioinformatics pipeline included adapter trimming and quality filtering of raw data, followed by alignment of reads to the GRCh38 reference genome using BWA-MEM v0.7.17 (http://bio-bwa.sourceforge.net/). Duplicate reads were marked using GATK MarkDuplicates v4.3.0.0 (McKenna et al., 2010). Single nucleotide variants and small indels were called using DeepVariant v4.0.0. For each sample, the fraction of reads with a quality score of at least Q30 was ≥90 % of the total reads obtained by sequencing.

Variant annotation was performed using Annovar (Wang et al., 2010). The clinical relevance of identified variants was assessed using the OMIM (http://www.omim.org/), ClinVar (https://www.ncbi.nlm.nih.gov/clinvar/), gnomAD v4.1.0 exomes (https://gnomad.broadinstitute.org/), HPO (https:// hpo.jax.org/), and Decipher (https://www.deciphergenomics. org/) databases, as well as data from the scientific literature

To validate the Xq13.1 microduplication, quantitative real-time PCR was performed on DNA from the proband and his parents using the following primer pairs (Syntol, Russia): targeting exon 2 of the EDA gene (F: 5ʹ-TGGCT ATGACTGAGTGGGGT-3ʹ; R: 5ʹ-GCTTCTTTTATTGC GGCGAAC-3ʹ) and targeting intron 1 of the EDA gene (F: 5ʹ-GTGGGATGTTGGAGGAACT-3ʹ; R: 5ʹ-TGCGAAC TTTGGTGAAGAAGC-3ʹ).

Primers were designed using the Primer-BLAST resource (https://www.ncbi.nlm.nih.gov/tools/primer-blast/). Selected primer sequences were tested for the formation of hairpins, self- and heterodimers using the OligoAnalyzer tool (https:// eu.idtdna.com/calc/analyzer).

The X-chromosome inactivation status was determined by amplifying a highly polymorphic CAG repeat in the first exon of the androgen receptor gene (AR, Xq12) after DNA hydrolysis with the methylation-sensitive restriction endonuclease HpaII. PCR products were separated using fragment analysis. An inactivation skewing of less than 80 % was considered a random pattern of X-chromosome inactivation, whereas a skewing of more than 80 % was regarded as indicative of skewed (asymmetric) X-chromosome inactivation (sXCI) (Allen et al., 1992).

## Results

Clinical case report. The proband, a 14-year-old male, was examined at the Genetic Clinic of the Research Institute of Medical Genetics, Tomsk National Research Medical Center, due to suspected anhidrotic ectodermal dysplasia. His medical history was significant for congenital absence of sweating and impaired thermoregulation. At the age of two months, he was diagnosed with atopic dermatitis and subsequently followed up for this condition, which was characterized by recurrent episodes of acute allergic manifestations, including atopic dermatitis and urticaria, requiring numerous hospitalizations in early childhood. His primary dentition erupted at 8 months of age and was notable for conical (awl-shaped) teeth.

The initial diagnosis of Christ–Siemens–Touraine syndrome with an X-linked recessive inheritance pattern was established at the age of 1 year and 5 months following a consultation by a clinical geneticist, based on the characteristic clinical presentation.

At the age of 2 years, the patient developed phimosis with the formation of synechiae. At 6 years, the proband was admitted to a surgical hospital with a diagnosis of acute balanoposthitis and preputial synechiae. From the age of 7 years, he has been followed up by a neurologist for an obsessivecompulsive disorder and a localized motor tic involving the periorbital musculature.

During a follow-up examination by a clinical geneticist at the age of 14, the following phenotypic features were noted: hypodontia of the upper jaw with conical teeth, anodontia of the lower jaw, hypotrichosis, periorbital hyperpigmentation, and palmoplantar keratoderma. The patient was subsequently referred for molecular genetic confirmation of the diagnosis.

Whole-genome sequencing identified a hemizygous ~397.3 kb duplication on chromosome X, spanning approximate coordinates chrX:69,620,777–70,018,072 and affecting the disease‑associated EDA gene (Fig. 1).

**Fig. 1.**
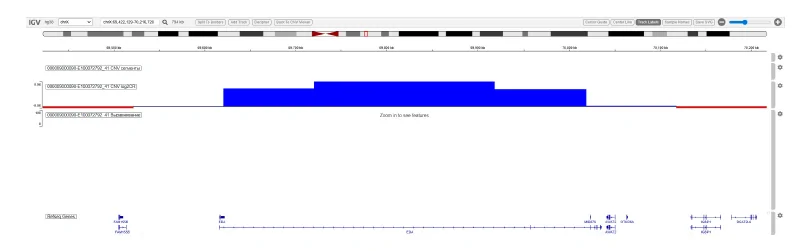
Proband NGS sequencing (IGV browser).

The Xq13.1 microduplication was confirmed in the proband and his mother by quantitative real-time PCR (Fig. 2). Neither of the proband’s parents exhibited any clinical manifestations of the disease.

**Fig. 2.**
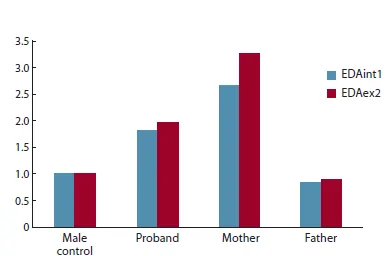
Copy number analysis of the Xq13.1 region in the proband and his parents. Results of real-time PCR assays targeting intron 1 and exon 2 of the EDA gene. The X-axis represents the male control sample, proband, mother, and father; the Y-axis shows the relative DNA copy number change.

The proband’s parents showed no clinical manifestations of the disease. Analysis of the X-chromosome inactivation status in the proband’s mother demonstrated random X-chromosome inactivation (74/26 %).

## Discussion

The EDA gene spans 419 kb, comprises eight exons, and encodes ectodysplasin A, a signaling ligand of the tumor necrosis factor (TNF) family, which plays a key role in the formation of ectodermal structures during embryogenesis. The function of ectodysplasin A is to transmit a positive signal supporting cell survival, proliferation, and differentiation during epithelialmesenchymal interactions, particularly in the development of hair follicles (Laurikkala et al., 2002).

According to the literature, hypohidrotic ectodermal dysplasia is frequently associated with atopic conditions, including atopic dermatitis, allergic rhinitis, and bronchial asthma (Stein, 2007). This association may be explained by impaired barrier function of the skin and mucous membranes, leading to xerosis (dry skin), as well as by thermoregulatory dysfunction resulting from sweat gland abnormalities. Dysfunction of bronchial glands has also been reported, which may predispose individuals to recurrent respiratory infections and bronchitis. In our clinical case, the patient showed signs of atopic dermatitis from the first months of life, requiring medical management.

The clinical presentation in the proband is consistent with the classical phenotype of X-linked hypohidrotic ectodermal dysplasia (Itin, Fistarol, 2004). In addition, the clinical features are similar to previously reported cases associated with pathogenic copy number variations (CNVs) affecting the EDA gene, including both deletions (Patient-1, Patient-2) and a duplication (Patient-3) (see the Table).

**Tab. 1.**
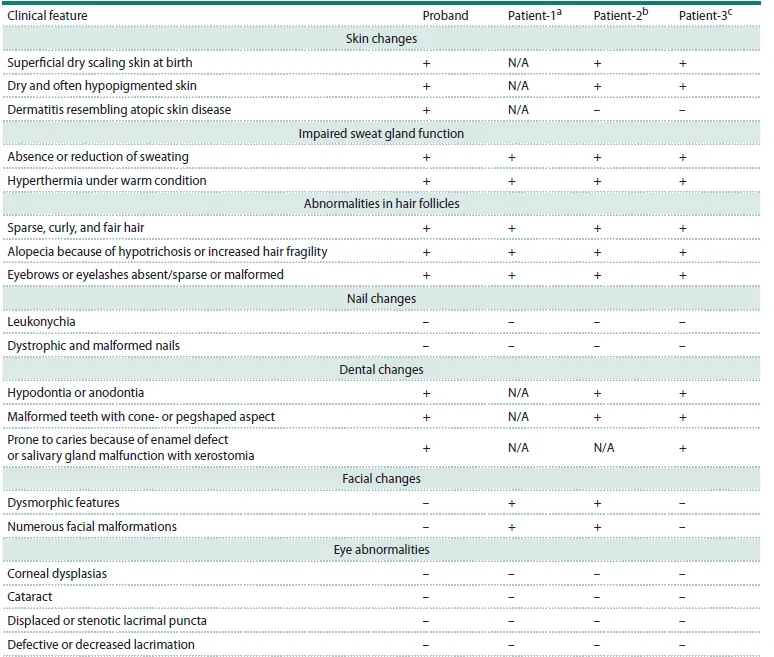
Comparison of clinical features of the proband with literature data Note. Sources: a (Hayashi et al., 2013); b (Li et al., 2008); c (Al Marzouqi et al., 2014); N/A – Not Assessed.

According to the literature and genomic databases, three cases of intragenic copy number variations (CNVs) have been described to date: two microdeletions and one microduplication. All of these cases were reported in patients presenting with a pronounced clinical picture consistent with the specific disease phenotype.

In Patient-1 (Hayashi et al., 2013), a deletion was identified and designated as NC_000023.11(NM_001399.5):c.397- 1589_502+13848del. This rearrangement corresponds to an intragenic deletion within the Xq13.1 region, spanning genomic coordinates chrX:69955438-69970980 and involving exon 2 of the EDA gene. In Patient-2 (Li et al., 2008), a 22-bp intragenic deletion was identified within exon 8 of the EDA gene, resulting in a frameshift. In Patient-3 (Al Marzouqi et al., 2014), an intragenic duplication of EDA exon 2 was reported. In addition, a duplication with similar coordinates is registered in the Decipher database (Patient:289747) and classified as pathogenic (Fig. 3).

**Fig. 3.**
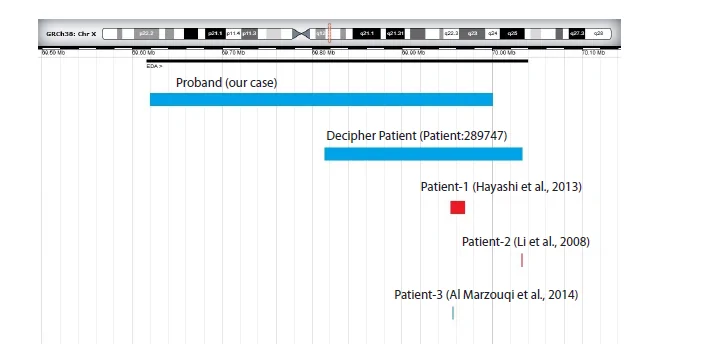
Schematic scale-fitted representation of rearrangements in previously reported patients with duplications (blue bars), deletions (red bars), and the proband within the EDA gene.

Structural variants with similar coordinates are not observed in the population frequency databases gnomAD CNVs/ SVs v4.1.0.

According to the ACMG criteria established for the assessment of CNV pathogenicity, this rearrangement is classified as pathogenic, receiving a score of 1.0 (criteria 1A, 2A, 3A) (Riggs et al., 2020). Based on the refined ACMG/AMP criteria adapted for the evaluation of monogenic CNVs, this rearrangement is also classified as pathogenic (PVS1, PM2, PP4) (Brandt et al., 2020).

X-linked hypohidrotic ectodermal dysplasia is known to exhibit incomplete penetrance in female carriers due to its X-linked recessive inheritance pattern (Munoz et al., 1997; Cambiaghi et al., 2000). The identified variant was inherited from the mother, who is a heterozygous asymptomatic carrier. Clinical manifestations of X-linked recessive disorders in women may be absent due to the heterozygous state of the X chromosome and random X-chromosome inactivation (Vincent et al., 2001). However, if skewed X-chromosome inactivation is present, female carriers may exhibit clinical symptoms when the homologue carrying the pathogenic variant remains active. In this case, we found random X-chromosome inactivation in the proband’s mother, which explains the absence of clinical manifestations of the disease. This rearrangement is assessed as pathogenic based on a combination of clinical data, pathogenicity criteria, and literature reports

An important aspect of the presented clinical case is the utilization of whole-genome sequencing for the diagnosis of this disease. Verifying an intragenic microduplication on the X chromosome in a male patient using standard NGS approaches (such as targeted gene sequencing or whole-exome sequencing) is extremely challenging. In a male, the hemizygous state of the X chromosome, combined with a copy number gain in a specific region, could be misinterpreted as increased gene coverage, potentially leading to a false negative diagnosis. Furthermore, methods designed to determine DNA copy number, such as MLPA, array CGH, or real-time PCR, are not routinely used in the diagnosis of Christ–Siemens– Touraine syndrome, because pathogenic variants are represented by various single nucleotide variants (SNVs) in up to 90 % of cases.

## Conclusion

This report presents a clinical case of X-linked hypohidrotic ectodermal dysplasia caused by a previously unreported ~397.3 kb duplication at Xq13.1, affecting the EDA gene. The duplication breakpoints map to approximately chrX:69,620,777–70,018,072. The diagnosis was established based on the characteristic clinical presentation. Molecular genetic confirmation allowed us to clarify the etiology and identify carrier status in the asymptomatic mother with random X-chromosome inactivation. This finding enables preconception and prenatal diagnostic options for future pregnancies in this family, given the potential for transmission by female carriers.

## Conflict of interest

The authors declare no conflict of interest.
